# Alginate/Hydroxyapatite-Based Nanocomposite Scaffolds for Bone Tissue Engineering Improve Dental Pulp Biomineralization and Differentiation

**DOI:** 10.1155/2018/9643721

**Published:** 2018-08-02

**Authors:** Silvia Sancilio, Marialucia Gallorini, Chiara Di Nisio, Eleonora Marsich, Roberta Di Pietro, Helmut Schweikl, Amelia Cataldi

**Affiliations:** ^1^Department of Pharmacy, University G. d'Annunzio, Chieti-Pescara, Italy; ^2^Department of Medical, Oral and Biotechnological Sciences, University G. d'Annunzio, Chieti-Pescara, Italy; ^3^Department of Conservative Dentistry and Periodontology, University Hospital Regensburg, University of Regensburg, Regensburg, Germany; ^4^Department of Medical, Surgical and Health Sciences, University of Trieste, Trieste, Italy; ^5^Department of Medicine and Ageing Sciences, G. d'Annunzio University, Chieti, Italy

## Abstract

Tissue engineering is widely recognized as a promising approach for bone repair and reconstruction. Several attempts have been made to achieve materials that must be compatible, osteoconductive, and osteointegrative and have mechanical strength to provide a structural support. Composite scaffolds consisting in biodegradable natural polymers are very promising constructs. Hydroxyapatite (HAp) can support alginate as inorganic reinforcement and osteoconductive component of alginate/HAp composite scaffolds. Therefore, HAp-strengthened polymer biocomposites offer a solid system to engineer synthetic bone substitutes. In the present work, HAp was incorporated into an alginate solution and internal gelling was induced by addition of slowly acid-hydrolyzing D-gluconic acid delta-lactone for the direct release of calcium ions from HAp. It has been previously demonstrated that alginate-based composites efficiently support adhesion of cancer bone cell lines. Human dental pulp stem cells (DPSCs) identified in human dental pulp are clonogenic cells capable of differentiating in multiple lineage. Thus, this study is aimed at verifying the mineralization and differentiation potential of human DPSCs seeded onto scaffolds based on alginate and nano-hydroxyapatite. For this purpose, gene expression profile of early and late mineralization-related markers, extracellular matrix components, viability parameters, and oxidative stress occurrence were evaluated and analyzed. In summary, our data show that DPSCs express osteogenic differentiation-related markers and promote calcium deposition and biomineralization when growing onto Alg/HAp scaffolds. These findings confirm the use of Alg/HAp scaffolds as feasible composite materials in tissue engineering, being capable of promoting a specific and successful tissue regeneration as well as mineralized matrix deposition and sustaining natural bone regeneration.

## 1. Introduction

Tissue engineering is widely recognized as one of the most promising approaches for bone repair and reconstruction in orthopaedics [1]. Bone is a complex and hierarchical tissue with nano-hydroxyapatite and collagen representing the major portions [2]. Several attempts have been made in order to achieve feasible materials with osteoconductive and osteointegrative properties to provide a structural support [3]. Indeed, the major role of bone is to provide a morphological framework, mechanical strength, blood pH regulation, and maintenance of calcium and phosphate levels to assist metabolic processes [2]. As far as dental restoration is concerned, regenerative endodontics is a promising field of tissue engineering that is aimed at achieving results using stem cells associated with responsive molecules and scaffolds. In particular, the combination of composite materials and stem cells in the site of implantation is considered an ideal alternative treatment to traditional root canal therapy [4]. Thus, growing cells from a small biopsy, followed by their culture in porous scaffold to form a new tissue, is the goal of tissue engineering [3]. Artificial scaffolds are temporary structures designed to sustain and promote cell adhesion, migration, proliferation, and differentiation through their chemical and physical properties [2]. Both bioactive ceramics and polymers have been developed for use as bone composite scaffolds. Composite scaffolds consisting in biodegradable natural polymers are very promising constructs, endowed with excellent biocompatibility and suitable mechanical properties. Moreover, they can be loaded with growth and differentiation factors involved in bone formation. Natural polymers offer the advantage of being very similar to the physiological macromolecular environment of cells [1, 5]. Among natural polymers, polysaccharides are very versatile, able to be decorated with signal molecules and to interact with inorganic components. Alginates belong to a family of linear copolymers capable of forming stable gels in the presence of millimolar concentrations of calcium or other divalent cations [6]. Alginates can be effectively manufactured into porous three-dimensional biodegradable networks, and, in combination with hydroxyapatite (HAp) as inorganic reinforcement and osteoconductive element and/or with other bioactive components, they can constitute solid systems sustaining natural bone regeneration [1, 7, 8].

Over the last decade, the discovery of different types of multipotent stem cells from human adult tissues has led to a rapid breakthrough in the field of regenerative medicine. They can potentially regenerate different types of tissues when loaded on suitable scaffolding structures that direct stem cell fate [9]. Adult mesenchymal stem cells (MSCs), primarily isolated from the bone marrow, were identified to be multipotent stem cells with the ability of differentiating into cells of mesodermal (osteocytes, chondrocytes, and adipocytes), ectodermal (neurons), and endodermal origins. Other eminent sources of adult MSCs include placenta, umbilical cord, amniotic fluid, adipose tissue, dental pulp, breast milk, and synovium [10]. The oral cavity as well as the extracted teeth constitute a remarkable source since they produce a variety of somatic stem cells [11]. The major differences in the stem cells arising from different sources are exhibited in their immunophenotypes. These differences have implications on proliferation, differentiation, and immunomodulation of MSCs from the respective sources. Dental pulp stem cells (DPSCs) have been reported to have higher proliferative ability than bone marrow- and adipose tissue-derived MSCs [10, 12–14]. They continuously divide and differentiate into various cell types, for instance, cells belonging to osteogenic, dentinogenic, adipogenic, neurogenic, chondrogenic, and myogenic lineage. Moreover, dental pulp can be easily isolated from the surgical waste derived from deciduous tooth or wisdom tooth extraction [15]. The very low morbidity of the anatomical site after the collection of the pulp, the high efficiency of the extraction procedure of stem cells from the pulp tissue, the differentiation ability, and the demonstrated interactivity of stem cells derived from dental pulp with biomaterials for tissue engineering applications hold to DPSCs a great potential for clinical purposes [11, 16]. Although it has been well demonstrated that MSCs from various sources are able to sustain bone regeneration [17], only recent tissue engineering approaches have shown that DPSCs constitute a valuable cell source for the healing of bone tissue when supported by a suitable scaffold [18–20].

Also compared to equal volumes of bone marrow, dental pulp contains a higher number of mesenchymal stem cells [21, 22]. In this light, due to the presence of a higher undifferentiated population, DPSCs are a suitable stem cell source in order to obtain different kinds of tissue including dental pulp tissues, dentin, or bone. These cells proliferate and differentiate into mature cells that produce an extracellular mineralized matrix. Remarkably, it seems that redox homeostasis might be essential for osteogenic differentiation [23]. MSCs are known to have low levels of intracellular ROS and high levels of glutathione, a key antioxidant. They also constitutively express high levels of enzymes required to manage oxidative stress. In terms of redox regulation, numerous recent reports describe the importance of oxidants on MSC differentiation into osteoblast-like cells. Elevated levels of ROS, defined as oxidative stress, lead to arrest of the MSC cell cycle and apoptosis [23, 24].

The differentiation stage of DPSCs greatly influences both the speed of matrix deposition and the quality of the newly formed one [22, 25]. Among several components, water with some dissolved noncollagenous organic matter is the third component of the mineralized matrix, while the ratios of hydroxyapatite-to-collagen-to-water volume and weight fraction are not constant. Wet collagen and hydroxyapatite mineral crystal agglomerations interpenetrate each other, forming the mineralized fibril at a scale of several hundred nanometers [2]. The aim of this study is to verify the mineralization potential of human DPSC growth in the presence of composite scaffolds based on alginate and nano-hydroxyapatite, which has been already described and characterized by Turco et al. [1]. It has been shown that these scaffolds can efficiently sustain adhesion, colonization, and matrix deposition of osteoblast-like cells without any additional chemical modification of alginate. Moreover, they have adequate structural and physical-chemical properties for being used as scaffolds in bone tissue engineering strategies. In the present paper, the gene expression profile of early and late mineralization-related markers, extracellular matrix components, viability parameters of DPSCs, and expression of bone sialoprotein II (BSP II) were evaluated and analyzed. We further provide experimental evidence that the activation of the enzymatic antioxidant catalase is crucial for cell survival.

## 2. Materials and Methods

### 2.1. Preparation of Alginate and HAp Composites (Alg/HAp Scaffolds)

Alg/HAp composite scaffolds were prepared after having mixed alginate 2% (*w*/*v*) and HAp at different concentrations in water using the calcium release method. HAp in powder was homogenously dissolved into a stirred solution of alginate in water, followed by the addition of GDL 60 mM to release calcium ions from HAp. Aliquots of this gel were then pipetted and incubated in a 24-well tissue culture plate (Costar, Cambridge, MA) for 24 h at room temperature in order to achieve a complete gelification. Hydrogels were then stepwise cooled, immersing tissue culture plates in a liquid cryostat using an aqueous solution of ethylene glycol (3 : 1 in water) as refrigerant fluid. Temperature was lowered stepwise from 20 up to −20°C using 5°C steps with a 30 min interval for equilibration. Samples were then freeze-dried for 24 h to obtain porous scaffolds. As a control, pure alginate gels (HAp-free) were prepared by replacing HAp with CaCO_3_ (30 mM of Ca^2+^). Hap-free gels were then processed as their counterpart with HAp. Alg/HAp scaffolds were further characterized as previously described [1].

### 2.2. Alg/HAp Scaffold Sterilization and Preparation for Cell Culture

Alg/HAp scaffolds underwent two cycles of sterilization under a UV light (15 W) for 1 hour. After that, they were rehydrated in a sterile aqueous solution of 5 mM CaCl_2_ and 1% penicillin/streptomycin under weak magnetic agitation for 20 minutes. Next, every scaffold was gently squeezed by means of a clamp and placed in a Falcon® 12-Well Clear Flat Bottom Not Treated Multiwell Cell Culture Plate. MEM ALPHA medium supplemented with 10% FBS and 1% penicillin/streptomycin (all purchased by EuroClone, Milan, Italy) was immediately added up to the brim of each well in order to cover up the entire scaffold surface. Then scaffolds were conditioned by the medium overnight, placing the multiwell plate at 37°C and 5% CO_2_.

### 2.3. Isolation and Cultivation of DPSCs

According to the Italian Legislation and the code of ethical principles for medical research involving human subjects of the World Medical Association (Declaration of Helsinki), young donors who underwent extraction of the third molar signed an informed consent. This project has received the approval of the Local Ethical Committee of the University of Chieti-Pescara (approval number 1173, date of approval 31/03/2016). After having obtained dental pulp samples from surgical procedures, DPSCs were handled and cultivated as previously described [22].

DPSCs were then analyzed by flow cytometry in order to characterize the cells by their immunophenotype. Molecular markers commonly applied to identify mesenchymal stem cells were detected as described in a previous work from our group [26].

### 2.4. Cell Seeding on Alg/HAp Scaffolds

After being characterized by flow cytometry, DPSCs were cultured and expanded in MEM ALPHA medium supplemented with 10% FBS and 1% penicillin/streptomycin (all purchased by EuroClone, Milan, Italy) up to passage 7. Cell cultures were then trypsinized (Trypsin/EDTA 1x, EuroClone, Milan, Italy), and cells were collected by centrifugation at 1200 rpm for 10 minutes at room temperature. DPSCs were counted by Trypan blue dye exclusion, and 5 × 10^4^ cells resuspended in 130 *μ*l of complete MEM ALPHA medium were used for cell seeding on each scaffold. The same cell number was used to seed DPSCs on polystyrene (named in figures as DPSCs). After having discarded the medium from the overnight conditioning, scaffolds were once again squeezed to eliminate the excess of medium and placed on a fresh Falcon 12-Well Clear Flat Bottom Not Treated Multiwell Cell Culture Plate. Next, DPSCs were seeded drop by drop on the scaffolds and immediately placed at 37°C and 5% CO_2_ for 3 h. Finally, a complete MEM ALPHA medium was added (named in figures as “Scaffold”, i.e., DPSC-laden scaffolds). Where necessary, the complete MEM ALPHA medium supplemented by differentiation agents (named in figures as DPSCs + DM and Scaffold + DM, i.e., DPSC-laden scaffolds in the presence of differentiation medium) was added. Complete DM was supplemented by L-ascorbic acid 100 *μ*M, dexamethasone 10 nM, *β*-glycerol phosphate disodium salt pentahydrate 5 mM (all purchased by Sigma-Aldrich, MI, USA), and potassium phosphate monohydrate 1.8 mM (Alfa Aesar, MA, USA). DPSCs seeded on polystyrene and DPSCs onto Alg/HAp scaffolds with or without DM were incubated for 1, 3, 7, 14, 21, and 28 days, refreshing the medium every 72 h.

### 2.5. Alizarin Red S Staining

To assess calcium deposition, Alizarin Red S staining was carried out on DPSC cultures. On days 7, 14, 21, and 28, the cell supernatant was discarded and cells were washed twice with PBS with Ca/Mg. After that, cultures were fixed with 4% paraformaldehyde (PFA) for 30 minutes and then washed with PBS. Samples were afterwards incubated with Alizarin Red S (40 mM in deionized water) for 30 minutes in the dark. After that, DPSCs were washed three times with deionized water and samples were observed under a phase-contrast light microscope (Leica, Wetzlar, Germany) equipped with a CoolSNAP camera to acquire computerized images (Photometrics, Tucson, AZ).

### 2.6. Alkaline Phosphatase Activity

Alkaline phosphatase (ALP) catalyzes the hydrolysis of phosphate esters in alkaline buffer and produces an organic radical and inorganic phosphate. The activity of ALP was analyzed in cell supernatants using the Alkaline Phosphatase Assay Kit (Colorimetric) (Abcam, Cambridge, UK). The kit uses *p*-nitrophenyl phosphate (pNPP) as a phosphatase substrate which turns yellow (*λ*_max_ = 405 nm) when dephosphorylated by ALP. Cell supernatants were collected after 1, 3, 7, 14, 21, and 28 days of culture. After collection, 80 *μ*l/well of sample was loaded in a Falcon 96-Well Clear Flat Bottom TC-Treated Culture Microplate in duplicate. Next, 50 *μ*l of *p*NPP 5 mM/well was added and the plate was incubated for 1 hour at room temperature in the dark. After that, 20 *μ*l of stop solution was pipetted into each well and the absorbance output was measured at 405 nm by means of a microplate reader (Multiskan GO, Thermo Scientific, MA, USA). Each test was performed in triplicate. The calculation of ALP activity (U/l/min) was carried out following the manufacturer's specifications, and each obtained value was normalized on its protein content (*μ*g of proteins/sample) measured by the BCA assay.

### 2.7. Quantification of Human Collagen Type I

Levels of collagen type I in the culture medium were quantified by means of ELISA (human collagen type 1 ELISA, Cosmo Bio, Tokyo, Japan). After 1, 3, 7, 14, 21, and 28 days, cell supernatants were collected and ELISA assay was performed. Cell supernatants were pipetted, and the blue conjugate and the yellow antibody were added. After 2 h of incubation at room temperature on a plate shaker, wells were washed and then incubated with the pNPP substrate for 45 min. Finally, a stop solution was added and optical density (OD) was measured at 450 nm by means of a microplate reader (Multiskan GO, Thermo Scientific, MA, USA). Each test was performed in triplicate. The concentration of collagen type I was calculated using a standard curve generated with specific standards provided by the manufacturers. Each obtained collagen concentration (*μ*g/ml) was normalized on the protein content (*μ*g of proteins/sample) measured by the BCA assay.

#### 2.8. Protein Extraction and BCA Assay

After having discarded the supernatants, DPSCs cultured on polystyrene were washed twice with PBS, trypsinized, and collected by centrifugation at 1200 rpm for 10 minutes. Pellets were then washed twice with cool PBS and kept on ice. As regard DPSCs' growth onto scaffolds, they were put in a sodium citrate buffer solution at pH = 7.4 under vigorous magnetic agitation in order to decompose scaffolds without damaging cells. This buffer solution was made by 50 mM sodium citrate tribasic dehydrate, 100 mM sodium chloride, and 10 mM sucrose (all purchased from Sigma-Aldrich, MI, USA). After being dissolved in the buffer solution, scaffolds and DPSCs were collected by centrifugation at 1500 rpm for 10 minutes and pellets were washed twice with cool PBS. After having discarded the washing solution, 0.5 ml of lysis buffer enriched with a protein inhibitor cocktail (PBS, 1% IGEPAL CA-630, 0.5% sodium deoxycholate, 0.1% SDS, 10 mg/ml PMSF, 1 mg/ml aprotinin, 100 mM sodium orthovanadate, and 50 *μ*g/ml leupeptin, all purchased from Sigma-Aldrich, MI, USA) was added and samples were kept on ice for 30 min. Then, the lysed pellets were resuspended and kept on ice for a further 30 min. Following centrifugation for 15 min at 20,000*g*, the supernatant was collected as the whole cell fraction. Protein concentration in the whole cell lysate was determined using bicinchoninic acid assay (QuantiPro™ BCA Assay Kit for 0.5–30 *μ*g/ml protein, Sigma-Aldrich, Milan, Italy) following the manufacturer's instructions. Briefly, cell lysates were diluted 1 : 150 in 150 *μ*l of deionized water and an equal volume of QuantiPro BCA assay mix was added. Samples were incubated 2 h at 37°C, and the absorbance signal generated was measured at 562 nm in a microplate reader (Multiskan GO, Thermo Scientific, MA, USA). Each test was performed in triplicate. Analyses of the obtained OD were carried out using GraphPad Prism version 5.01 for Windows (GraphPad Software, San Diego, CA). The concentration of protein was calculated using a standard curve generated with specific standards provided by the manufacturers.

### 2.9. Cytotoxicity Assay

Cytotoxicity occurrence was assessed by means of the CytoTox 96® Non-Radioactive Cytotoxicity Assay (Promega, WI, USA). The kit measures lactate dehydrogenase (LDH), a stable cytosolic enzyme that is released upon cell lysis. Released LDH in culture supernatants is measured with coupled enzymatic assay, which results in the conversion of a tetrazolium salt (iodonitrotetrazolium violet (INT)) into a red formazan product. The amount of color formed is proportional to the number of lysed cells. After having collected supernatants at days 1, 3, 7, 14, 21, and 28, 50 *μ*l was transferred to a Falcon 96-Well Clear Flat Bottom TC-Treated Culture Microplate in triplicate. An equal volume of CytoTox 96 Reagent is added to each well and incubated for 30 minutes. When a provided stop solution was added, the absorbance signal was measured at 490 nm and 690 nm (background) in a microplate reader (Multiskan GO, Thermo Scientific, MA, USA). Each test was carried out in triplicate. Assessment of the percentage of relative cytotoxicity was calculated subtracting the background value and normalizing the obtained OD with the protein content (*μ*g of proteins/sample) measured by the BCA assay.

### 2.10. Catalase Activity

To indirectly assess oxidative stress occurrence, the analysis of the antioxidant enzyme catalase activity was carried out. For this purpose, the Amplex® Red Catalase Assay Kit was used (Molecular Probes, Invitrogen Corporation, CA, USA). Catalase is a heme-containing redox protein which prevents excess of intracellular hydrogen peroxide (H_2_O_2_) converting this compound to water and oxygen. In the assay, catalase—if working in samples—first reacts with H_2_O_2_ to produce water and oxygen (O_2_). Next, the Amplex Red reagent reacts with a 1 : 1 stoichiometry with any unreacted H_2_O_2_ in the presence of horseradish peroxidase (HRP) to produce the highly fluorescent oxidation product, resorufin. Therefore, as catalase activity increases, the signal from resorufin decreases. The absorbance was measured at 560 nm in a microplate reader (Multiskan GO, Thermo Scientific, MA, USA). The results are typically plotted by subtracting the observed fluorescence from that of a no-catalase control (complete MEM ALPHA medium and complete medium with DM). Assessment of the relative catalase activity (mU/ml) was calculated using a standard curve generated with specific standards provided by the manufacturers and normalizing the obtained values with the protein content (*μ*g of proteins/sample) measured by the BCA assay.

### 2.11. RNA Extraction

Total RNA was extracted using TRI Reagent (Sigma-Aldrich, St. Louis, MO). Briefly, cell growth onto Alg/HAp scaffolds was suspended in 500 *μ*l of TRI Reagent and then centrifuged at 10,000 rpm for 10 min at 4°C. The supernatant was added to 100 *μ*l of chloroform, then shaken vigorously, incubated on ice for 15 min, and centrifuged at 13,200 rpm for 20 min at 4°C. RNA in aqueous phase was precipitated with 250 *μ*l of isopropanol, stored for 30 min at −20°C, and pelletted by centrifugation at 13,200 rpm for 20 min at 4°C. RNA pellet was washed with 500 *μ*l of 75% ethanol, air-dried, and finally resuspended in RNAse-free water. In order to avoid the contamination of samples, DNA was removed using a DNA-free kit (Life Technologies, Carlsbad, CA). RNA concentration was determined by spectrophotometer reading at 260 nm, and its purity was assessed by the ratio at 260 and 280 nm readings (BioPhotometer Eppendorf, Hamburg, Germany). Samples were afterwards tested by electrophoresis through agarose gels and visualized by staining with ethidium bromide, under UV light.

### 2.12. Reverse Transcription (RT) and Real-Time RT-Polymerase Chain Reaction (Real-Time RT-PCR)

In order to reverse-transcribe 1 *μ*g of RNA, a high-capacity cDNA Reverse Transcription Kit (Life Technologies) was used. Twenty microliters of these solutions was stepwise incubated in a 2720 Thermal Cycler (Life Technologies) initially at 25°C for 10 min, then at 37°C for 2 h and finally at 85°C for 5 min. Gene expression was determined by quantitative PCR using TaqMan probe-based chemistry. Reactions were performed in 96-well plates on an ABI PRISM 7900 HT Fast Real-Time PCR System (Life Technologies). TaqMan probes and PCR primers were obtained from Life Technologies (TaqMan Gene Expression Assays (20x): Hs00154192_m1 for BMP2, Hs00231692_m1 for RUNX2, and Hs01866874_s1 for SP7), and glyceraldehyde 3-phosphate dehydrogenase (GAPDH) (Life Technologies, part number 4333764 F) was used as a housekeeping gene. Each amplification reaction was performed with 10 *μ*l of TaqMan Fast Universal PCR Master Mix (2x), No AmpErase UNG (Life Technologies), 1 *μ*l of primer-probe mixture, 1 *μ*l of cDNA, and 8 *μ*l of nuclease-free water. No-template control was used to check for contamination. A reverse transcriptase minus control was included for SP7 gene assay. Thermal cycling conditions were 95°C for 20 s, followed by 40 cycles of amplification at 95°C for 1 s and 60°C for 20 s. Sequence Detection System software, ver. 2.3 (Life Technologies), elaborated gene expression data. The comparative 2^−ΔΔCt^ method was used to quantify the relative abundance of mRNA (relative quantification). Real-time PCR analysis was performed in three independent experiments. In each experiment, we included one cDNA sample for each experimental condition. Amplification was carried out in triplicate for each cDNA sample in relation to each of the investigated genes.

### 2.13. Immunoblotting

DPSC lysates (20 *μ*g/sample) were electrophoresed on a 4–20% SDS-PAGE gel (ExpressPlus™ 10x8, GenScript Biotech Corporation, China) and transferred to nitrocellulose membranes further blocked in 5% of nonfat milk, 10 mmol/l Tris pH 7.5, 100 mM NaCl, and 0.1% Tween 20. Membranes were then probed for mouse anti-*β*-actin monoclonal antibody (Sigma-Aldrich, St. Louis, MO, USA) (primary antibody dilution 1 : 10,000) and mouse monoclonal anti-BSP II (Santa Cruz Biotechnology, CA, USA) (primary antibody dilution 1 : 200) and incubated in the presence of specific conjugated IgG horseradish peroxidase. Immunoreactive bands were identified using the ECL detection system (Amersham Int., Buckinghamshire, UK) and analyzed by densitometry. Densitometric values, expressed as the percentage of the integrated optical intensity ratio of BSP II and *β*-actin, were estimated by the ChemiDoc XRS system using the QuantiOne 1-D analysis software (Bio-Rad, Richmond, CA, USA).

### 2.14. Statistics

Statistical analysis was performed using the analysis of variance (one-way ANOVA) with post hoc test (Tukey) for RT-PCR analysis and the *t*-test. Results were expressed as mean ± SD. Values of *p* < 0.05 were considered statistically significant.

## 3. Results

### 3.1. Formation of Mineralized Nodules in DPSCs Exposed to Differentiation Medium

**Figure 1:**
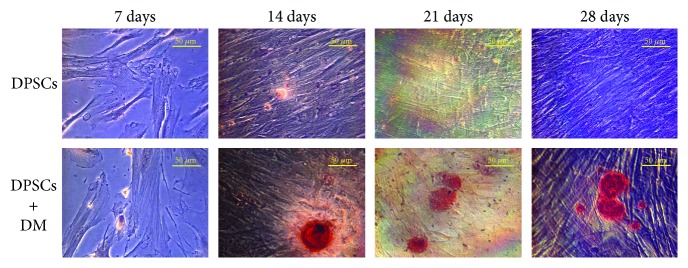
Mineralization of dental pulp stem cells (DPSCs) assessed by Alizarin Red staining. The upper panel shows the Alizarin Red staining of pulp cells after 7, 14, 21, and 28 days of culture without differentiation medium (DM). The presence of mineralized nodules in cultures exposed to DM after 7, 14, 21, and 28 days is displayed in the lower panel. Magnification 40x.

The production of the extracellular mineralized matrix by DPSCs was evaluated through Alizarin Red S staining. As shown in Figure 1, cells cultured in the presence of differentiation agents (DM) are crowded by a mineralized matrix rich in calcium precipitates stained in red (lower panel), revealing that the generation of calcium precipitates is time-dependent. Quite the opposite, cells from the control group show fewer Alizarin Red-positive regions starting from 7 days of culture up to 28 days (upper panel).

### 3.2. Alkaline Phosphatase Activity and Collagen Type I Release in DPSCs Exposed to Medium Enriched with Mineralizing Agents

**Figure 2:**
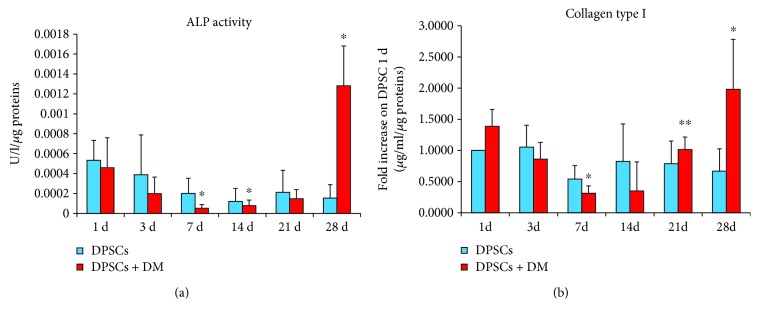
Alkaline phosphatase (ALP) activity and collagen type I release in dental pulp stem cells (DPSCs). (a) Bar graph showing the enzymatic activity of ALP (U/ml) normalized on the protein content (*μ*g/sample) after 1, 3, 7, 14, 21, and 28 days of culture. ^∗^ *p* < 0.05, 7, 14, and 28 d of DPSCs + DM versus DPSCs. (b) The bar graph represents collagen type I secretion from DPSCs with or without DM after 1, 3, 7, 14, 21, and 28 days of culture. Results are normalized on values obtained from DPSCs cultured for 1 day without DM after being proportioned on the protein content (*μ*g/sample). ^∗^ *p* < 0.05 28 d DPSCs + DM versus DPSCs. ^∗∗^ *p* < 0.01 21 d DPSCs + DM versus DPSCs.

In order to evaluate mineralization occurrence and to confirm Alizarin Red S staining observation, ALP activity was quantified in DPSCs committed to osteogenic differentiation (DPSCs + DM) compared to cells cultivated in normal growth medium (DPSCs) (Figure 2(a)). After 1 day of culture, only a slight difference among DPSCs and DPSCs + DM in terms of ALP activity is detectable (5.33 × 10^−4^ U/l/min and 4.6 × 10^−4^ U/l/min, resp.). As far as DPSC culture is concerned, ALP activity decreases in a time-dependent manner, being assessed at 3.88 × 10^−4^ U/l/min after 3 days of culture and at 1.54 × 10^−4^ U/l/min after 28 days. A similar tendency can be observed for DPSCs exposed to DM, being significantly dropped after 7 and 14 days (5.13 × 10^−5^ U/l/min and 7.75 × 10^−5^ U/l/min, resp.). Cells exposed to differentiation agents for 28 days produce the highest peak in terms of ALP activity, being significantly increased compared to DPSCs cultivated in normal growth medium (1.28 × 10^−3^ U/l/min versus 1.54 × 10^−4^).

After that, collagen type I secretion was quantified to evaluate the extracellular matrix modifications and the osteogenic commitment (Figure 2(b)). As regard DPSCs cultivated in normal growth medium, after a slight decrease occurred up to 7 days, the levels of secreted collagen type I arise again to the level of the first day of culture. In parallel, cells exposed to differentiation agents show a decrease in collagen type I production reaching the lowest values after 7 and 14 days (0.311 *μ*g/ml and 0.349 *μ*g/ml). After 21 days, this tendency is reversed and the production of collagen type I is significantly higher with respect to the first day of culture (1.98 *μ*g/ml).

### 3.3. Protein Quantification and Cytotoxicity

**Figure 3:**
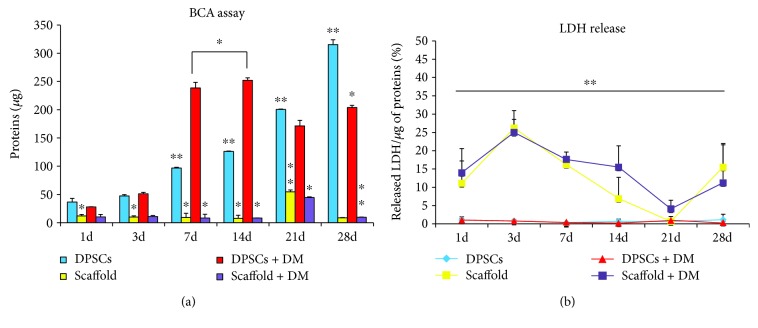
Protein content and cytotoxicity assay on dental pulp stem cells (DPSCs) in the presence of alginate/hydroxyapatite scaffolds. (a) The bar graph represents the quantification of protein content (*μ*g/sample) measured by the bicinchoninic acid (BCA) assay of DPSCs in the presence of alginate/hydroxyapatite scaffolds over the different experimental conditions. ^∗^ *p* < 0.05 7, 14, and 28 d DPSCs + DM versus DPSCs; 1, 3, 7, and 14 d scaffold versus DPSCs; 7, 14, and 21 d scaffold + DM versus DPSCs + DM. ^∗∗^ *p* < 0.01 7, 14, 21, and 28 d DPSCs versus 1 d DPSCs; 21 d scaffold versus DPSCs; 28 d scaffold + DM versus scaffold. (b) Lactate dehydrogenase (LDH) released from DPSCs seeded on alginate/hydroxyapatite scaffolds with or without DM. ^∗∗^ *p* < 0.01 1, 3, 7, 14, 21, and 28 d scaffold versus DPSCs; 1, 3, 7, 14, 21, and 28 d scaffold + DM versus DPSCs + DM. Scaffold = DPSC-laden scaffolds; scaffold + DM = DPSC-laden scaffolds in the presence of differentiation medium.

As a measure of cell seeding efficiency, the BCA assay was performed on the whole cell lysates derived from DPSCs cultivated onto Alg/HAp scaffolds compared to the ones from DPSCs under normal cell conditions with or without differentiation agents (Figure 3(a)). As regards control group (DPSCs), the protein content gradually arises over the time of the experiments, being almost 9-fold augmented after 28 days compared to 1 and 3 days of culture (315.17 *μ*g, 36.57 *μ*g, and 47.40 *μ*g, resp.). A similar tendency can be observed for DPSCs cultivated in the presence of differentiation agents up to 14 days when the absolute highest protein content is registered (27.97 *μ*g, 51.18 *μ*g, 238.45 *μ*g, and 251.87 *μ*g). After that, a slight decrease occurs, with the protein content assessed at 171.35 *μ*g after 21 days and at 203.84 *μ*g after 28 days. Although this fall is significant compared to 14 and 7 days of culture, the amount of proteins remains consistent with respect to early stages (Figure 3(a)). When DPSCs were cultivated onto Alg/HAp scaffolds, the protein pool is significantly reduced compared to DPSCs alone after 1 day of culture (11.95 *μ*g and 36.57 *μ*g, resp.). The shrinkage in terms of protein content can be observed up to 14 days, when the absolute lower value is registered (7.64 *μ*g). Surprisingly, a 7-fold increase is detected (54.61 *μ*g) after 21 days of culture. Nevertheless, the protein pool is once again lowered after 28 days. A similar tendency can be registered when DPSCs are cultivated onto scaffolds in the presence of differentiation agents (scaffold + DM). For instance, the amount of protein after 21 days, when there is the peak for DPSCs and scaffold, is even lower (44.57 *μ*g).

After having monitored cell seeding efficiency by means of protein quantification, the cytotoxicity of Alg/HAp scaffolds on DPSCs was assessed in terms of LDH release (Figure 3(b)). There are no significant changes in the percentage of LDH released from cells exposed to DM or cultivated alone, as the highest peaks for these experimental conditions are assessed at 1.18% after 28 days of culture of DPSCs alone. Quite the opposite, a substantial elevation in the percentage of LDH released can be detected when DPSCs are grown onto Alg/HAp scaffolds. More in detail, the amount of enzyme secreted after 1 day is 11-fold higher than the one released from DPSCs alone (11.06% versus 1.06%). After 3 days of cell culture on the scaffold, this rise is even more enhanced, reaching a 32.7-fold increase if compared to DPSCs alone (26.20% versus 0.80%). After this peak, the percentage of LDH released gradually decreases, being assessed at 0.68% after 21 days of culture (Figure 3(b)). Once again, a rise in terms of LDH release is observed after 28 days of culture (15.57%). A similar progress can be registered when DPSCs are cultivated onto scaffolds in the presence of DM, with the exception of the 14 days of culture, when the percentage of LDH is quite higher with respect to DPSCs and scaffold alone (15.52% versus 6.93%).

### 3.4. Formation of Oxidative Stress

**Figure 4:**
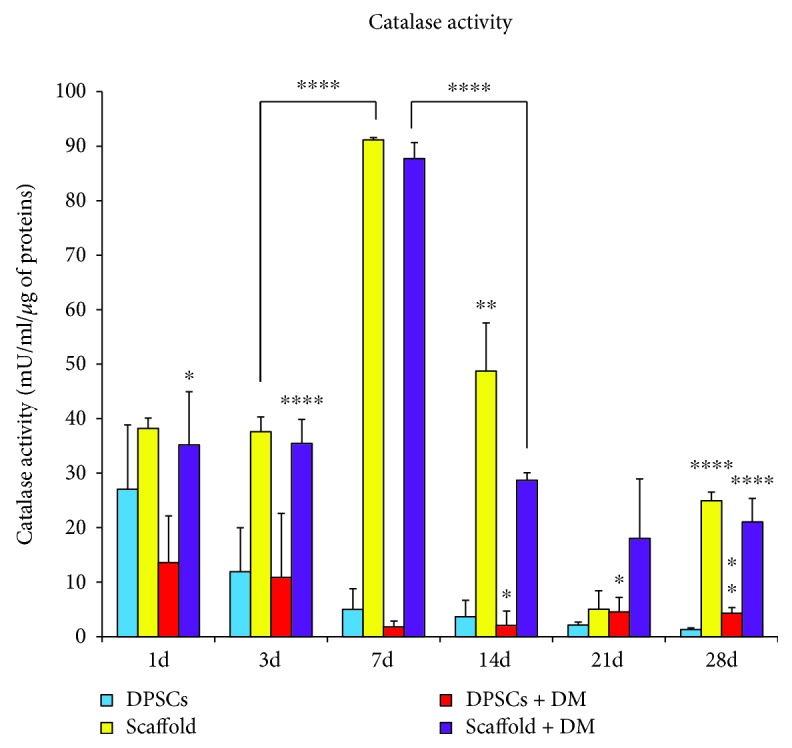
Catalase activity of dental pulp stem cells (DPSCs) in the presence of alginate/hydroxyapatite scaffolds. Bars display the modulation of catalase activity (mU/ml) normalized on the protein content (*μ*g/sample) of DPSCs over the different experimental conditions. ^∗^ *p* < 0.05 14 and 21 d DPSCs + DM versus DPSCs; 1 d scaffold + DM versus DPSCs + DM. ^∗∗^ *p* < 0.01 28 d DPSCs + DM versus DPSCs; 14 d scaffold versus DPSCs. ^∗∗∗∗^ *p* < 0.005 14 d scaffold + DM versus scaffold; 3, 7, and 28 d scaffold versus DPSCs; 3, 7, 14, and 28 d scaffold + DM versus DPSCs + DM. Scaffold = DPSC-laden scaffolds; scaffold + DM = DPSC-laden scaffolds in the presence of differentiation medium.

To correlate the elevated cytotoxicity to an oxidative stress occurrence, the activity of catalase was monitored and quantified (Figure 4). When DPSCs are cultured alone, the activity of the antioxidant enzyme gradually decreases, being 27.04 mU/ml after 1 day of culture and 1.31 mU/ml after 28 days. Very likely, when DPSCs are exposed to DM, the activity of catalase lowers with respect to DPSCs without DM up to 14 days of culture. After 21 days of culture in the presence of DM, catalase activity is doubled with respect to DPSCs alone (4.54 mU/ml versus 2.14 mU/ml) and the difference is even more enhanced after 28 days (4.31 mU/ml versus 1.31 mU/ml). Observing catalase activity in DPSCs growth onto scaffolds, a dramatic rise can be detected compared to both DPSCs exposed to DM and DPSCs alone (Figure 4). More in detail, values appear similar as regard 1 and 3 days of culture (38.21 mU/ml and 37.58 mU/ml, resp.). At 7 days of culture onto scaffolds, the absolute highest value of catalase activity is registered (91.12 mU/ml). After that peak, the activity of the enzyme progressively decreases, reaching 24.94 mU/ml after 28 days. When DM is added to the DPSCs/scaffold culture, the tendency results to be very similar to the previous one. The catalase activity after 7 days is slightly lower (87.70 mU/ml) but still consistent compared to DPSCs + DM. As above, the activity of the enzyme lessens, being 21.05 mU/ml after 28 days of culture.

### 3.5. Gene Expression Profile of Mineralization-Related Markers

**Figure 5:**
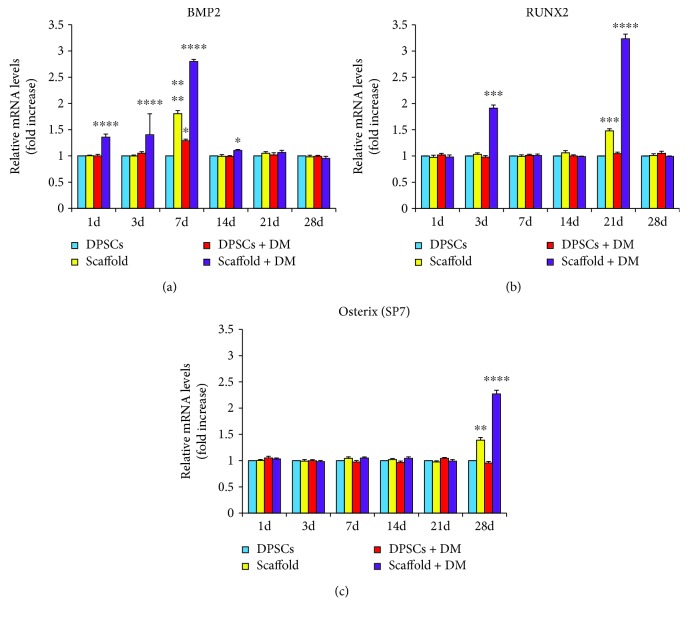
Gene expression profiles of differentiation- and mineralization-associated marker genes in dental pulp stem cells (DPSCs) in the presence of alginate/hydroxyapatite scaffolds. Graphs represent the relative gene expression of BMP2 (a), RUNX2 (b), and Osterix (SP7) (c) in DPSCs over the various experimental conditions. Data are expressed as fold increase on relative mRNA levels of DPSCs cultivated in growth medium not enriched with differentiation agents. (a) ^∗^ *p* < 0.05 14 d scaffold + DM versus DPSCs; 7 d DPSCs + DM versus DPSCs. ^∗∗∗∗^ *p* < 0.001 7 d scaffold versus DPSCs; 1, 3, and 7 d scaffold + DM versus DPSCs. (b) ^∗∗∗^ *p* < 0.005 21 d scaffold versus DPSCs; 3 and 21 d scaffold + DM versus DPSCs. (c) ^∗∗^ *p* < 0.01 28 d scaffold versus DPSCs. ^∗∗∗∗^ *p* < 0.001 scaffold + DM versus DPSCs. Scaffold = DPSC-laden scaffolds; scaffold + DM = DPSC-laden scaffolds in the presence of differentiation medium.

To demonstrate that a commitment to mineralization is established, relative gene expression of BMP2, RUNX2, and SP7 was analyzed by RT-PCR on DPSCs alone and growth onto Alg/HAp scaffolds along with their counterpart exposed to DM (Figure 5). At length, the expression of the early differentiation gene BMP2 (Figure 5(a)) is not significantly changed after 3 days of cell culture in the presence of DM. Notably, there is a consistent elevation of BMP2 when DPSCs are exposed to DM for 7 days. After that, values revert to the ones of the control group. When cells grow onto scaffolds, a significant positive peak is registered after 7 days of culture (1.805 fold increase). As regard DPSCs/scaffold culture + DM, the expression of BMP2 is dramatically increased earlier than 7 days, being elevated already after 1 day of culture and even more after 3 and 7 days (1.357-, 1.402-, and 2.798-fold increase, resp.).

Next, gene expression of RUNX2 was analyzed, which is found expressed here at later stages than BMP2 (Figure 5(b)). For instance, there is a climax after 21 days when DPSCs are grown onto scaffold (1.479-fold increase) with respect to both DPSCs alone and DPSCs + DM. When cells are cultivated in the presence of the scaffold and exposed to DM, two positive pulses of RUNX2 gene expression can be registered. The first one is after 3 days of culture, and the second—more dramatic—is observed after 21 days (1.910- and 3.233-fold increase, resp.).

Finally, the expression of the late differentiation gene SP7 was evaluated. Generally speaking, there are no significant differences up to 21 days of culture. After 28 days, the expression of SP7 is consistently enhanced when DPSCs are cultivated onto scaffolds (1.390-fold increase) and this tendency is even more evident when DM is added to the DPSC/scaffold model (2.272-fold increase).

### 3.6. ALP Activity and Collagen Type I Release in DPSC Growth onto Alg/HAp Scaffolds

**Figure 6:**
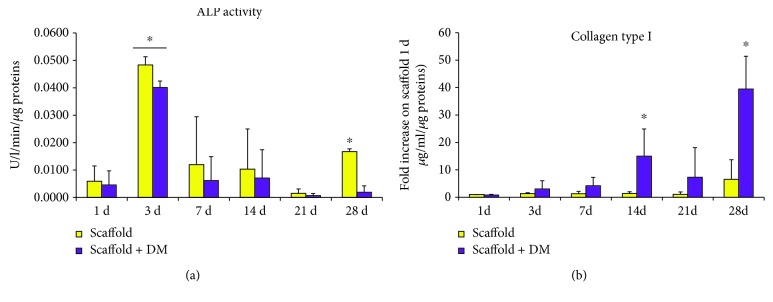
Alkaline phosphatase (ALP) activity and collagen type I release in dental pulp stem cells (DPSCs) in the presence of alginate/hydroxyapatite scaffolds. (a) Bar graph showing the enzymatic activity of ALP (U/ml) normalized on the protein content (*μ*g/sample) after 1, 3, 7, 14, 21, and 28 days of culture of DPSC growth onto scaffolds. ^∗^ *p* < 0.05 3 and 28 d scaffold versus 1 d scaffold; 3 d scaffold + DM versus 1 d scaffold + DM. (b) The bar graph represents collagen type I secretion from DPSCs cultured on scaffolds with or without DM after 1, 3, 7, 14, 21, and 28 days. Results are normalized on values obtained from DPSCs cultured on scaffolds for 1 day without DM after being proportioned on the protein content (*μ*g/sample). ^∗^ *p* < 0.05 14 and 28 d scaffold + DM versus scaffold and scaffold + DM. Scaffold = DPSC-laden scaffolds; scaffold + DM = DPSC-laden scaffolds in the presence of differentiation medium.

Finally, to further confirm DPSCs' osteogenic commitment when cultured onto Alg/HAp scaffolds in the presence or not of DM, the activity of ALP and the release of collagen type I were monitored (Figure 6). The presence of DM does not influence ALP activity after 1 day of culture, with U/l/min being very similar to the one of DPSCs on scaffold alone (Figure 6(a)). A positive peak can be observed when DPSCs are cultured onto scaffolds for 3 days. As deduced from the bar graph, this is the highest absolute value registered, being assessed at 0.0493 U/l/min. When DM is added to the culture, ALP activity slightly lowers, but remaining consistent (0.0401 U/l/min). After 7, 14, and 21 days of culture, there are only weak fluctuations of ALP activity, while it is quite elevated after 28 days when DPSCs are cultured onto scaffolds without DM.

In parallel, the concentration of collagen type I in cell supernatants was evaluated comparing all the values to the concentration after 1 day of culture of DPSCs on scaffold without DM (Figure 6(b)). A time-dependent concentration increase can be detected for DPSC growth onto scaffolds, with the release of collagen 1.32-fold higher after 3 days and 6.50-fold higher after 28 days with respect to 1 day of culture. Very likely but even more consistently, the secretion of collagen type I when DPSCs are grown onto scaffolds in the presence of DM is time-dependently increased. Indeed, the release of collagen after 28 days is 13-fold higher if compared to the one after 3 days (3.01-fold versus 39.46-fold, resp.).

### 3.7. BSP II Expression in DPSCs Seeded on HAp/Alg Scaffolds

**Figure 7:**
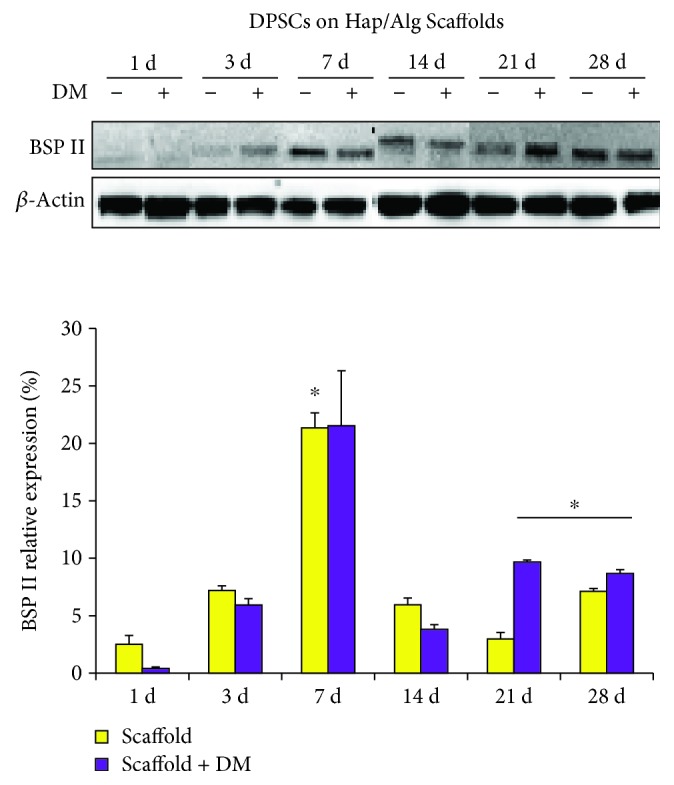
Bone sialoprotein II (BSP II) protein expression in dental pulp stem cells (DPSCs) in the presence of alginate/hydroxyapatite scaffolds by Western blotting analysis. Graph represents densitometric values (%) of BSP II expression normalized on *β*-actin expression. ^∗^ *p* < 0.05 7 days scaffold versus 1 day scaffold; ^∗^ *p* < 0.05 21 and 28 days scaffold + DM versus 1 day scaffold + DM. Scaffold = DPSC-laden scaffolds; scaffold + DM = DPSC-laden scaffolds in the presence of differentiation medium.

To confirm the osteogenic differentiation of DPSCs grown onto scaffolds in the presence or not of DM, the expression of the noncollagenous protein BSP II, implied in the regulation of bone mineralization, was analyzed by means of Western blotting. Densitometric values show a peak after 7 days of culture, registering a significant increased value when DPSCs are grown onto scaffolds without DM (Figure 7). After that, BSP II is notably expressed after 21 and 28 days when the DM is present.

## 4. Discussion

DPSCs isolated from human postnatal dental pulp tissues can give rise to multilineage differentiation *in vitro* and generate related mineralized tissues *in vivo* [27]. Bone and dentine extracellular matrix proteins are similar, consisting primarily of type I collagen, acidic proteins, and proteoglycans. Although collagen forms the lattice for deposition of calcium and phosphate assisting formation of carbonate apatite, noncollagenous proteins are believed to control initiation and growth of the crystals [28]. For this purpose, Alizarin Red S staining was carried out on our system. More in detail, DPSCs cultured on polystyrene exposed to medium enriched by differentiation agents show calcium nodule deposition from 7 days of culture compared to cells from the control group (Figure 1). The dimension of calcium nodules arises in a time-dependent manner in DPSCs cultured in the presence of DM with respect to DPSCs' growth in normal medium. In parallel, we evaluated ALP activity and secretion of collagen type I in the same experimental conditions. Despite different fluctuations, both parameters increased at late stages of culture, for instance, at 28 days. This is coherent with previous studies and literature [29], and the authors of the present work are quite confident on the functionality of the experimental system in committing cells to mineralized matrix deposition.

For most regenerative strategies, an organic scaffold is used to provide a surface on which cells may adhere, grow, and spatially organize [30]. In the present paper, a 3D biodegradable porous scaffold prepared from a binary mixture composed by alginate and HAp was used. DPSCs have to interpenetrate this porous structure and proliferate to give birth to newly produced mineralized tissue. Due to the difficult handling of this experimental 3D cell culture and the difficulty in obtaining data on cell seeding efficiency and viability with classical and commercially available assays, we measured the amount of proteins to indirectly evaluate DPSC attachment and interpolation into scaffolds. Not surprisingly, DPSCs better adhered on polystyrene, with the amount of proteins increased over the time of the culture. In the DPSC/scaffold system, an increment in the amount of protein was consistent after 21 days. In parallel, the protein pool was slightly lowered when DM is added. This let us speculate that DPSCs were more proliferating on scaffolds alone. Next, to evaluate the cytotoxic response of DPSCs on Alg/HAp scaffolds, the LDH assay was carried out. Observing in parallel the amount of proteins and the release of LDH, it is plausible to assume that nonproliferating DPSCs in the presence of scaffolds fit on necrotic cell death, especially after 3 days of culture. Quite the opposite, the LDH release, which gradually lowered from 7 days up to 21 days, let us hypothesize a compensatory mechanism. Data on LDH release agreed with the rise in protein amount at 21 days, when the release of LDH is very low and very close to the one of the control group (Figure 3). The hypothesized compensatory mechanism seems to default after 28 days in the same experimental conditions when a rise of LDH released and a decrease in the protein amount is registered. Most relevantly, DM seems to even enhance the reduction of the protein amount and rise of LDH release as evidenced by bar and trend graphs after 14 days of culture.

Hydrogen peroxide scavenging is governed by catalase, which is the enzyme that converts H_2_O_2_ in H_2_O and O_2_ [31]. To correlate the dramatic LDH release from DPSCs cultured onto scaffolds for 3 days and the recovering of the system after 7 days and on, we evaluated the activity of the antioxidant enzyme catalase (Figure 4). As shown by the bar graph, oxidative stress was not occurring in both DPSCs from the control group and the one exposed to DM, with the activity of catalase being physiologically compatible. On the other way round, the activity of catalase in DPSCs cultivated on Alg/HAp scaffolds was consistent already from early stages. Indeed, after 7 days, the highest absolute value is registered, making plausible the hypothesis that DPSCs produced a large amount of H_2_O_2_ when growing onto scaffolds. To counteract oxidative stress occurrence, cells respond by activating antioxidant molecules; among them, catalase is one of the terminal regulators [32]. Redox homeostasis is crucial for osteogenic commitment, differentiation, and cell survival [23]. Likewise, here DPSCs escaped from necrotic cell death, through the activation of catalase, as evidenced by the decrement of LDH released and the massive increase of catalase activity after 7 days (Figures 3 and 4).

The tissue engineering goal is not only cell proliferation and the lack of cell cytotoxic responses against the foreign material, but overall the production of newly born tissues and then stem cell commitment to osteogenic differentiation. Data from literature has demonstrated that seven days after H_2_O_2_ treatment, DPSCs showed significant reduction in ALP activity compared with negative control and no mineralized nodule deposition [34]. Hence, the evaluation of the gene profile of mineralization-related markers was mandatory. We therefore analyzed the expression of BMP2, RUNX2, and Osterix (SP7). The gene expression of BMP2 is recognized to stimulate mineralization, and it is considered the most osteogenic bone morphogenetic protein which implies the early induction of differentiation [35]. RUNX2 is an important transcriptional modulator of differentiation, maturation, and homeostasis, downstream from the BMP2 differentiation pathway [36]. In turn, Osterix (SP7) is a transcription factor which plays a pivotal role during the differentiation of progenitors into mature cells in a step downstream of RUNX2 [37]. In our system, expression of early, intermediate, and late differentiation markers is consistent with data in literature [22]. More in detail, BMP2 expression increased after 3 and 7 days in DPSCs cultured on scaffolds indifferently from the presence of DM, in accordance with data of catalase activity. Beside a spike after 3 days, RUNX2 was enhanced after 21 days mostly when DM is present, in agreement with LDH release after the same experimental time. As regards Osterix gene expression, it was clearly increased after 28 days mainly in DPSCs/scaffolds + DM, when the LDH amount was slightly decreased with respect to scaffolds alone (Figure 5). Several studies have reported the synthesis of many scaffolds capable of sustaining pulp stem cell differentiation *in vitro* in the presence of differentiation media, but only two of them, namely, a polyvinyl alcohol- (PVA-) poly(*ε*)caprolactone- (PCL-) hydroxyapatite-based bioceramic [38] and a calcium phosphate cement scaffold loaded with iron oxide nanoparticles [39], have been found to enhance cell differentiation compared to cell monolayers cultured on polystyrene due to the osteoinductive property of the material.

To confirm if DPSC enhanced mineralization when cultured on Alg/HAp scaffolds, we reevaluated ALP activity and collagen type I secretion. Data supported gene expression results, with ALP activity being strongly enhanced after 3 days of culture where DM was not influencing the activity of the enzyme. This tendency was similar over the experimental time, up to 28 days of culture (Figure 6(a)). Conversely, the release of collagen type I seemed to be consistently influenced by DM as shown in Figure 6(b). It is plausible to assume that this enhancement could be ascribed to the presence of ascorbic acid present in the DM medium. This is in agreement with data on literature which correlates the release of collagen and extracellular matrix deposition with exposures to ascorbate [40]. Finally, to strengthen DPSC's ability to differentiate into osteoblast-like cells, the expression of BSP II was analyzed. Among all, the dentin-bone noncollagenous proteins consist in small integrin-binding ligand N-linked glycoproteins (SIBLINGs). SIBLINGs are secreted during the formation and mineralization of both dentin and bone, and BSP is a highly posttranslationally modified protein that is an abundant noncollagenous component of the bone matrix. Moreover, in situ hybridization and immunolocalization experiments have shown that BSP is expressed in teeth during mineralization [41–43]. Our data show that BSP II is highly expressed after 7, 21, and 28 days on DPSCs grown onto scaffolds in the presence of DM in line with PCR results.

In summary, we provide evidence for osteoconductivity of alginate/hydroxyapatite scaffolds that are able of efficiently sustaining adhesion, colonization, and matrix deposition of osteoblast-like cells. [1]. We show that DPSCs express mineralization-related markers and proteins, definitely promoting matrix deposition. Most importantly, this physiological function is here related to redox homeostasis controlled by the activation of catalase which as an enzymatic antioxidant enhances cell survival.

### 4.1. Limitations of the Study

Although the cellular response towards oxidative stress is driven by a multidimensional network of transcription factors and enzymes and the measurement of catalase activity is only one aspect of the molecular scenario occurring intracellularly, catalase plays a crucial role in adaptive response to H_2_O_2_ and it has been suggested that its role is somehow auxiliary to that of glutathione peroxidase [33]. Furthermore, the differentiation of osteoblasts implies the formation of an extracellular matrix, which is rich in proteins. Due to the difficulty in the experimental design, in the handling of the three-dimensional cell culture and the specific properties of the material, to normalize ELISA and LDH results with the amount of protein**s** was the only appropriate experimental procedure because any other proliferation assays are not feasible.

## Data Availability

The data used to support the findings of this study are available from the corresponding author upon request.

## Supplementary Materials

Supplementary Materials S1. DPSC immunophenotyping: data reported show the representative immunophenotype profile of DPSCs isolated from the third molar at passage 4. Cells were found negative for the lymphocyte-related CD45 (protein tyrosine phosphatase) and slightly positive for SSEA4 (stage-specific embryonic antigen 4), while they significantly express CD90 (Thy-1), CD73 (5′ nucleotidase), CD29 (integrin *β*1), and CD105 (endoglin), thus identifying a mesenchymal stem cell population.
