# Supplementary material for: Alginate/Hydroxyapatite-Based Nanocomposite Scaffolds for Bone Tissue Engineering Improve Dental Pulp Biomineralization and Differentiation
Source: Stem Cells Int. 2018 Aug 2;2018:9643721. doi: 10.1155/2018/9643721 (PMC6098856; doi:10.1155/2018/9643721)
Supplement: Supplementary Materials — S1. DPSC immunophenotyping: data reported show the representative immunophenotype profile of DPSCs isolated from the third molar at passage 4. Cells were found negative for the lymphocyte-related CD45 (protein tyrosine phosphatase) and slightly positive for SSEA4 (stage-specific embryonic antigen 4), while they significantly express CD90 (Thy-1), CD73 (5′ nucleotidase), CD29 (integrin β1), and CD105 (endoglin), thus identifying a mesenchymal stem cell population. [file 9643721.f1.pdf]

Characterization DPSCs-Passage 4

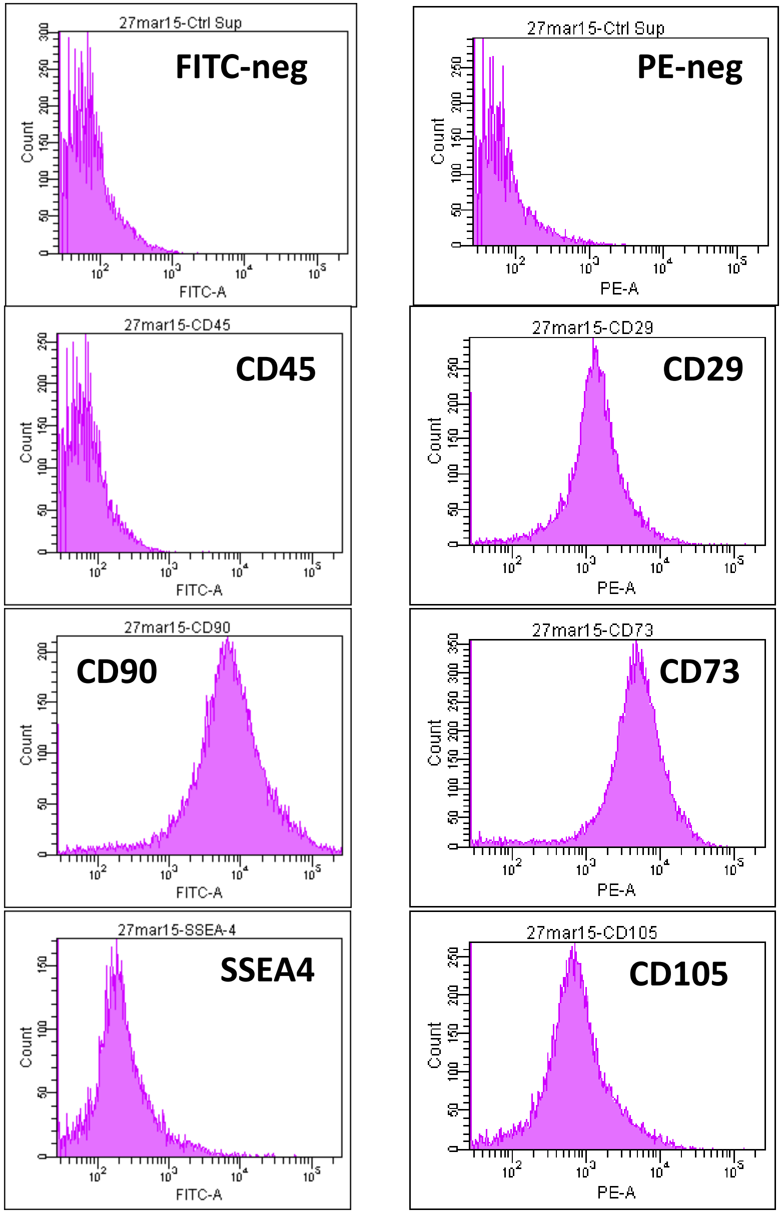

Immunophenotype (FACS)

CD45^{-} CD90^{++} SSEA4^{+}
CD29^{+} CD73^{++} CD105^{+}
